# Supplementary material for: Sublethal concentrations of undissociated acetic acid may not always stimulate acid resistance in Salmonella enterica sub. enterica serovar Enteritidis Phage Type 4: Implications of challenge substrate associated factors
Source: PLoS One. 2020 Jul 23;15(7):e0234999. doi: 10.1371/journal.pone.0234999 (PMC7377465; doi:10.1371/journal.pone.0234999)
Supplement: S1 Table — Different letters within columns at a given pH value indicate statistical differences among treatments at the same time intervals according to Tukey’s HSD test. (PDF) [file pone.0234999.s001.pdf]

Treatments	Time (min)						
	0	2.5	5	7.5	10	15	30
NA	5.3 ± 0.1	3.3 ± 0.5	0.8 ± 0.5	0.6 ± 0.5	-0.1 ± 0.4	-0.3 ± 0.0	-0.3 ± 0.0
0mM/pH6.0	5.7 ± 0.2 **(a)**	2.9 ± 1.1**(b)**	1.4 ± 0.6 **(b)**	1.0 ± 0.4 **(b)**	0.3 ± 0.7 **(c)**	0.1 ± 0.6 **(a)**	-0.2 ± 0.1
15mM/pH6.0	5.5 ± 0.1 **(b)**	3.8 ± 0.5 **(a)**	2.1 ± 0.5 **(b)**	1.2 ± 0.3 **(b)**	0.4 ± 0.7 **(ab)**	0.1 ± 0.4 **(a)**	-0.3 ± 0.1
25mM/pH6.0	5.4 ± 0.1 **(b)**	4.3 ± 0.4 **(a)**	2.0 ± 0.6 **(b)**	1.0 ± 0.4 **(b)**	0.3 ± 0.8 **(c)**	-0.2 ± 0.3 **(a)**	-0.3 ± 0.1
35mM/pH6.0	5.4 ± 0.1 **(b)**	4.6 ± 0.7 **(a)**	3.4 ± 0.9 **(a)**	2.0 ± 1.2 **(a)**	1.6 ± 1.1 **(ab)**	0.8 ± 0.8 **(a)**	-0.3 ± 0.1
45mM/pH6.0	5.3 ± 0.1 **(b)**	4.7 ± 0.2 **(a)**	3.4 ± 0.2 **(a)**	2.5 ± 0.3 **(a)**	1.8 ± 0.5 **(a)**	0.8 ± 0.7 **(a)**	-0.2 ± 0.3
0mM/pH5.5	5.7 ± 0.1 **(a)**	3.6 ± 0.9 **(b)**	1.8 ± 0.6 **(c)**	1.3 ± 0.7 **(b)**	1.4 ± 1.0 **(ab)**	0.7 ± 0.8 **(ab)**	-0,3 ± 0.0
15mM/pH5.5	5.2 ± 0.2 **(b)**	4.8 ± 0.4 **(a)**	3.5 ± 0.6 **(ab)**	2.4 ± 0.8 **(a)**	1.7 ± 0.6 **(a)**	0.6 ± 0.9 **(b)**	0.1 ± 0.5
25mM/pH5.5	5.1 ± 0.2 **(b)**	4.7 ± 0.3 **(a)**	3.5 ± 0.6 **(ab)**	2.7 ± 0.7 **(a)**	1.9 ± 0.6 **(a)**	0.4 ± 0.6 **(b)**	-0.3 ± 0.0
35mM/pH5.5	5.2 ± 0.1 **(b)**	4.8 ± 0.3 **(a)**	4.0 ± 0.7 **(a)**	3.2 ± 0.6 **(a)**	2.4 ± 0.6 **(a)**	1.7 ± 0.7 **(a)**	0.0 ± 0.4
45mM/pH5.5	5.2 ± 0.1 **(b)**	4.6 ± 0.2 **(a)**	2.6 ± 1.1 **(bc)**	1.3 ± 0.7 **(b)**	0.6 ± 0.6 **(b)**	0.0 ± 0.4 **(a)**	-0.2 ± 0.2
0mM/pH5.0	5.6 ± 0.1 **(a)**	3.8 ± 0.8 **(b)**	2.2 ± 0.5 **(a)**	1.4 ± 0.5 **(a)**	0.6 ± 0.7 **(a)**	0.3 ± 0.6 **(a)**	-0,3 ± 0.0
15mM/pH5.0	5.1 ± 0.2 **(b)**	4.4 ± 0.3 **(ab)**	2.6 ± 1.2 **(a)**	1.5 ± 0.7 **(a)**	0.8 ± 0.6 **(a)**	0.3 ± 0.5 **(a)**	-0,2 ± 0.2
25mM/pH5.0	4.9 ± 0.2 **(b)**	4.4 ± 0.4 **(ab)**	2.9 ± 0.6 **(a)**	1.4 ± 0.3 **(a)**	0.3 ± 0.6 **(a)**	-0.1 ± 0.4 **(a)**	-0.3 ± 0.1
35mM/pH5.0	5.0 ± 0.2 **(b)**	4.5 ± 0.5 **(a)**	2.9 ± 0.8 **(a)**	1.8 ± 1.0 **(a)**	1.0 ± 0.9 **(a)**	0.1 ± 0.4 **(a)**	-0.2 ± 0.2
45mM/pH5.0	5.0 ± 0.1 **(b)**	4.5 ± 0.1 **(a)**	2.9 ± 0.4 **(a)**	1.5 ± 0.4 **(a)**	07 ± 0.7 **(a)**	-0.3 ± 0.1 **(a)**	-0.3 ± 0.0
0mM/pH4.5	5.3 ± 0.1 **(a)**	4.6 ± 0.4 **(a)**	3.4 ± 0.9 **(a)**	2.0± 1.0 **(a)**	1.4 ± 1.0 **(a)**	0.3 ± 0.7 **(a)**	-0.2 ± 0.1
15mM/pH4.5	4.9 ± 0.1 **(b)**	4.6 ± 0.1 **(a)**	2.8 ± 0.4 **(a)**	1.1 ± 0.3 **(a)**	0.5 ± 0.7 **(a)**	0.1 ± 0.6 **(a)**	-0.2 ± 0.2
25mM/pH4.5	4.9 ± 0.2 **(b)**	4.6 ± 0.3 **(a)**	3.2 ± 0.5 **(a)**	1.4 ± 0.5 **(a)**	0.5 ± 0.5 **(a)**	-0.1 ± 0.4 **(a)**	-0.3 ± 0.1
35mM/pH4.5	4.9 ± 0.2 **(b)**	4.4 ± 0.4 **(a)**	3.3 ± 1.0 **(a)**	1.8 ± 1.0 **(a)**	1.0 ± 1.0 **(a)**	-0.2 ± 0.3 **(a)**	-0.3 ± 0.1
45mM/pH4.5	3.9 ± 0.2 **(b)**	4.4 ± 0.2 **(a)**	3.3 ± 0.4 **(a)**	1.7 ± 0.9 **(a)**	1.3 ± 0.7 **(a)**	0.6 ± 0.4 **(a)**	-0.3 ± 0.0
0mM/pH4.0	5.1 ± 0.2 **(a)**	4.6 ± 0.3 **(a)**	2.7 ± 1.0 **(a)**	1.3 ± 0.7 **(a)**	0.3 ± 0.8 **(a)**	-0.2 ± 0.3 **(a)**	-0.3 ± 0.0
15mM/pH4.0	5.0 ± 0.2 **(ab)**	4.5 ± 0.2 **(a)**	3.0 ± 0.5 **(a)**	1.2 ± 0.5 **(a)**	0.4 ± 0.4 **(a)**	0.3 ± 0.7 **(a)**	-0.3 ± 0.0
25mM/pH4.0	4.9 ± 0.2 **(ab)**	4.5 ± 0.4 **(a)**	3.2 ± 0.5 **(a)**	1.6 ± 0.7 **(a)**	0.3 ± 0.5 **(a)**	-0.3 ± 0.1 **(a)**	-0.3 ± 0.0
35mM/pH4.0	4.9 ± 0.2 **(ab)**	4.6 ± 0.3 **(a)**	3.3 ± 0.9 **(a)**	2.0 ± 1.1 **(a)**	1.2 ± 1.3 **(a)**	0.3 ± 0.6 **(a)**	-0.3 ± 0.1
45mM/pH4.0	4.9 ± 0.2 **(b)**	4.1 ± 0.2 **(b)**	2.4 ± 0.7 **(a)**	1.3 ± 0.6 **(a)**	0.5 ± 0.6 **(a)**	0.1 ± 0.3 **(a)**	-0.3 ± 0.0
