# Supplementary material for: Sublethal concentrations of undissociated acetic acid may not always stimulate acid resistance in Salmonella enterica sub. enterica serovar Enteritidis Phage Type 4: Implications of challenge substrate associated factors
Source: PLoS One. 2020 Jul 23;15(7):e0234999. doi: 10.1371/journal.pone.0234999 (PMC7377465; doi:10.1371/journal.pone.0234999)
Supplement: S2 Table — Different letters within the same row indicate statistical differences according to Tukey’s HSD test. (PDF) [file pone.0234999.s002.pdf]

	Treatment (log CFU/ml ± SD)			
Time (h)	Non-adapted	15mM/pH5.0	35mM/pH5.5	45mM/pH6.0
0	5.2 ± 0.1 **(b)**	5.1 ± 0.1 **(ab)**	5.2 ± 0.1 **(ab)**	5.3 ± 0.1 **(a)**
24	4.4 ± 0.2 **(b)**	4.6 ± 0.3 **(b)**	5.0 ± 0.2 **(a)**	5.2 ± 0.1 **(a)**
32	4.2 ± 0.3 **(c)**	4.3 ± 0.2 **(bc)**	4.5± 0.1 **(b)**	5.0 ± 0.1 **(a)**
48	3.2 ± 0.3 **(b)**	3.4 ± 0.3 **(b)**	3.5 ± 0.2 **(b)**	4.3 ± 0.3 **(a)**
56	2.1 ± 0.8 **(c)**	2.9 ± 0.3 **(b)**	2.8 ± 0.5 **(b)**	4.0 ± 0.3 **(a)**
72	1.0 ± 0.1 **(b)**	1.0 ± 0.0 **(b)**	1.0 ± 0.9 **(b)**	2.8 ± 0.7 **(a)**
96	1.0 ± 0.0 **(b)**	1.9 ± 0.0 **(b)**	1.0 ± 0.0 **(b)**	1.2 ± 0.3 **(a)**
