# Supplementary material for: Sublethal concentrations of undissociated acetic acid may not always stimulate acid resistance in Salmonella enterica sub. enterica serovar Enteritidis Phage Type 4: Implications of challenge substrate associated factors
Source: PLoS One. 2020 Jul 23;15(7):e0234999. doi: 10.1371/journal.pone.0234999 (PMC7377465; doi:10.1371/journal.pone.0234999)
Supplement: S3 Table — Different letters within the same row indicate statistical differences according to Tukey’s HSD test. (PDF) [file pone.0234999.s003.pdf]

	Treatment (log CFU/ml ± SD)			
Time (days)	Non-adapted	15mM/pH5.0	35mM/pH5.5	45mM/pH6.0
0	5.2 ± 0.1 **(a)**	5.2 ± 0.1 **(a)**	5.1 ± 0.1 **(a)**	5.3 ± 0.1 **(a)**
1	4.8 ± 0.4 **(b)**	4.9 ± 0.1 **(b)**	4.9 ± 0.1 **(b)**	5.1 ± 0.2 **(a)**
2	4.1 ± 0.1 **(b)**	4.2 ± 0.1 **(ab)**	4.3 ± 0.2 **(ab)**	4.5 ± 0.5 **(a)**
3	3.3 ± 0.1 **(c)**	3.4 ± 0.3 **(bc)**	3.6 ± 0.3 **(b)**	5.5 ± 0.4 **(a)**
4	2.0 ± 0.8 **(a)**	2.5 ± 0.7 **(a)**	2.7 ± 0.8 **(a)**	
5	1.4 ± 0.6 **(ab)**	1.5 ± 0.6 **(a)**	1.0 ± 0.0 **(b)**	
6	1.0 ± 0.0 **(a)**	1.1 ± 0.3 **(a)**	1.0 ± 0.0 **(a)**	
7	1.0 ± 0.0	1.0 ± 0.0	1.0 ± 0.0	
