# Supplementary material for: Sublethal concentrations of undissociated acetic acid may not always stimulate acid resistance in Salmonella enterica sub. enterica serovar Enteritidis Phage Type 4: Implications of challenge substrate associated factors
Source: PLoS One. 2020 Jul 23;15(7):e0234999. doi: 10.1371/journal.pone.0234999 (PMC7377465; doi:10.1371/journal.pone.0234999)
Supplement: S6 Table — Different letters within the same row indicate statistical differences according to Tukey’s HSD test. (PDF) [file pone.0234999.s006.pdf]

	Treatment (log CFU/ml ± SD)			
Time (days)	Non-adapted	15mM/pH5.0	35mM/pH5.5	45mM/pH6.0
0	6.4 ± 0.1 **(b)**	6.7 ± 0.1 **(a)**	6.8 ± 0.1 **(a)**	6.8 ± 0.1 **(a)**
6	6.0 ± 0.2 **(b)**	6.2 ± 0.2 **(ab)**	6.3 ± 0.1 **(a)**	6.0 ± 0.3 **(b)**
12	5.3 ± 0.3 **(b)**	5.6 ± 0.3 **(a)**	5.4 ± 0.3 **(ab)**	5.3 ± 0.2 **(ab)**
18	4.3 ± 0.7 **(a)**	4.4 ± 0.7 **(a)**	4.5 ± 0.5 **(a)**	4.3 ± 0.6 **(a)**
21	3.5 ± 0.9 **(a)**	3.8 ± 0.9 **(a)**	3.9 ± 0.8 **(a)**	3.8 ± 0.7 **(a)**
24	3.0 ± 1.1 **(a)**	2.9 ± 1.3 **(a)**	3.3 ± 1.3 **(a)**	3.2 ±1.1 **(a)**
27	3.0 ± 1.0 **(a)**	3.2 ± 1.4 **(a)**	3.4 ± 1.1 **(a)**	3.4 ± 1.4 **(a)**
30	2.6 ± 0.7 **(a)**	2.4 ± 0.7 **(a)**	2.7 ± 0.9 **(a)**	2.6 ± 1.0 **(a)**
33	2.0 ± 0.5 **(a)**	2.2 ± 0.8 **(a)**	2.1 ± 0.7 **(a)**	2.1 ± 0.6 **(a)**
35	1.7 ± 0.0 **(a)**	1.7 ± 0.0 **(a)**	1.9 ± 0.4 **(a)**	1.9 ± 0.4 **(a)**
37	1.7 ± 0.0	1.7 ± 0.0	1.7 ± 0.0	1.7 ± 0.0
