# Supplementary material for: Sublethal concentrations of undissociated acetic acid may not always stimulate acid resistance in Salmonella enterica sub. enterica serovar Enteritidis Phage Type 4: Implications of challenge substrate associated factors
Source: PLoS One. 2020 Jul 23;15(7):e0234999. doi: 10.1371/journal.pone.0234999 (PMC7377465; doi:10.1371/journal.pone.0234999)
Supplement: Attachment — Submitted filename: Response to reviewers.docx [file pone.0234999.s008.docx]

**Panagiotis N. Skandamis**

**Associate Professor**

**Agricultural University of Athens**

**118 55, Athens, Greece**

***E-mail:*** pskan@aua.gr

**Dr. Leonida Matsaka**

**Academic Editor**

**PLOS ONE**

June 1^st^, 2020

**Ref: Revision of Manuscript PONE-D-20-07602**

**“Studying the effect of undissociated acetic acid and pH on the acid resistance of *Salmonella enterica* sub. *enterica* serovar Enteritidis Phage Type 4: implications of food matrix associated factors”**

Dear Editor,

We would like to thank you for your decision to accept our manuscript to be revised. We have adopted the majority of the comments proposed by the referee. After careful adjustment of the text, we thank the reviewers for their comments that helped us to substantially improve the presentation of our work. Our reply to the Editor’s and reviewers’ comments is as follows:

- **Journal requirements**

*1. Please ensure that your manuscript meets PLOS ONE's style requirements, including those for file naming. The PLOS ONE style templates can be found at*

<https://journals.plos.org/plosone/s/file?id=wjVg/PLOSOne_formatting_sample_main_body.pdf> and

<https://journals.plos.org/plosone/s/file?id=ba62/PLOSOne_formatting_sample_title_authors_affiliations.pdf>

**Authors’ reply: In the revised version we complied carefully with all style requirements of the PLOS ONE journal.**

*2. In your Methods section, please provide additional details regarding each of the cell lines used in your study, including any quality control testing procedures, and ensure you have described the source. For more information regarding PLOS' policy on materials sharing and reporting, see* [*https://journals.plos.org/plosone/s/materials-and-software-sharing#loc-sharing-materials*](https://journals.plos.org/plosone/s/materials-and-software-sharing#loc-sharing-materials)*, and for more information on PLOS ONE's guidelines for research using cell lines, see* [*https://journals.plos.org/plosone/s/submission-guidelines#loc-cell-lines*](https://journals.plos.org/plosone/s/submission-guidelines#loc-cell-lines)*. Also please provide additional details regarding the food materials used in your study and ensure you have described the source.*

**Authors’ reply: Details regarding the source of provision of *Salmonella* Enteritidis strain alongside with the composition and source of food matrix (tarama salad) used in the current study were included in the Methods section. Please see lines 120-123, 210-217 of the marked copy and 100-103, 186-193 of the clear (umarked) copy.**

- **General Comments**

*1. Is the manuscript technically sound, and do the data support the conclusions?*

*The manuscript must describe a technically sound piece of scientific research with data that supports the conclusions. Experiments must have been conducted rigorously, with appropriate controls, replication, and sample sizes. The conclusions must be drawn appropriately based on the data presented.*

*Reviewer #1: Yes*

*Reviewer #2: Partly*

**Authors’ reply: All the sections of the manuscript were extensively revised in order to improve the presentation of our work based on the comments mentioned below. Tables 1 (kinetic parameter estimates of screening assay) and 2 (kinetic parameter estimates of tarama salad assay) as well as Fig 2 (4D_pH_/4D_NA_ ratio), were modified due to changes in the selection of the inactivation models. In any case, the trends remained the same.**

*2. Has the statistical analysis been performed appropriately and rigorously?*

*Reviewer #1: Yes*

*Reviewer #2: Yes*

**Authors’ reply: We thank both reviewers for their positive comments to our work.**

*3. Have the authors made all data underlying the findings in their manuscript fully available?

The* [*PLOS Data policy*](http://journals.plos.org/plosone/s/data-availability) *requires authors to make all data underlying the findings described in their manuscript fully available without restriction, with rare exception (please refer to the Data Availability Statement in the manuscript PDF file. The data should be provided as part of the manuscript or its supporting information, or deposited in a public repository. For example, in addition to summary statistics, the data points behind means, medians and variance measures should be available. If there are restrictions on publicly sharing data—e.g. participant privacy or use of data from a third party—these reasons must be specified.*

*Reviewer #1: Yes*

*Reviewer #2: Yes*

**Authors’ reply: We thank both reviewers for their positive comments to our work.**

*4. Is the manuscript presented in an intelligible fashion and written in standard English?

PLOS ONE does not copyedit accepted manuscripts, so the language in submitted articles must be clear, correct, and unambiguous. Any typographical or grammatical errors should be corrected at revision, so please note any specific errors here.*

*Reviewer #1: Yes*

*Reviewer #2: Yes*

**Authors’ reply: We thank both reviewers for their positive comments to our work.**

*5. Review Comments to the Author*

*Please use the space provided to explain your answers to the questions above. You may also include additional comments for the author, including concerns about dual publication, research ethics, or publication ethics. (Please upload your review as an attachment if it exceeds 20,000 characters)*

**Reviewer #1:**

*L54. Missing parenthesis;*

**Authors’ reply: The missing parenthesis was added (line 59 of the marked copy and 53 of the clear copy).**

*L108-109. Indicate the country of production of the acetic acid and sodium hydroxide;*

**Authors’ reply: The country of production of acetic acid (line 137 of the marked copy and 117 of the clear copy) and sodium hydroxide (line 139 of the marked copy and 118-119 of the clear copy) alongside with the rest microbiological/chemical materials used in the experimental procedure are indicated in the revised version.**

*L113. Should be deleted “or no growth’;*

**Authors’ reply: The phrase “or no growth” was deleted.**

*L185. Where is the detection limit known? Literature source?*

*Determine the inherent micro flora paragraph (Line 190-193) could be integrated in paragraph (173-186) or specified somewhere separately;*

**Authors’ reply: Τhe detection limit of a microbiological method is calculated as follows: by the volume (ml) of the dilution inoculated in the petri dishes and by the lowest dilution available for inoculation.**

**The “Determination of inherent microflora of tarama salad” paragraph was integrated in the subsection “Impact of adaptive responses in tarama salad or TSB adjusted to pH 4.35”. Please see lines 218-221 of the marked copy and 194-197 of the clear copy.**

*L306-307. Table 1. It should be noted that acetic acid buffers may be used??*

*At least half of the references should be updated from 2015-2020;*

**Authors’ reply: Table 1 title was modified so as to include the presence of acetic acid in some of the adaptive treatments tested. Please see lines 426-429 of the marked copy and 306-308 of the clear copy.**

**Acid adaptation has received wide attention the last decades, starting with the pioneer studies of primarily two teams, Foster (Foster, 1991, Foster and Hall, 1990; 1991) in *Salmonella* Typhimurium and Rowbury (Goodson and Rowbury, 1989) in *Escherichia coli*, at the early 1990’s. Since then, several studies have further established the ability of sublethal acid conditions to promote the survival of foodborne pathogens, covering a range of scientific issues. Nevertheless, to our knowledge, recent studies pertaining acid adaptation of these two pathogens are mainly focusing on molecular mechanisms by which *Salmonella* spp. and *E. coli* can sense and adapt to acidic environments. Therefore, the majority of the studies related to our work are dated mainly before 2010. In any case, after careful screening of the literature, we tried to replace – whenever possible- older references with more recent ones, and if available from the time period 2015-2020.**

- **Foster, John W. 1991. *Salmonella* acid shock proteins are required for the adaptive acid tolerance response. Journal of Food Bacteriology 173:21, 6896-6902**
- **Foster John W., Hall Holly K. 1990. Adaptive acidification tolerance response of *Salmonella typhimurium*. Journal of Food Bacteriology 172:2, 771-778**
- **Foster John W., Hall Holly K. 1991. Inducible pH homeostasis and the acid tolerance response of *Salmonella typhimurium.* Journal of Food Bacteriology 173:16, 5129-5135**
- **Goodson M. and Rowbury R. J. 1989. Habituation to normally lethal acidity by prior growth of *Escherichia coli* at a sub-lethal acid pH value. Letters in Applied Microbiology 8, 77-79**

**Reviewer #2:**

*This manuscript reports the effect of undissociated acetic acid on the induction of cell acid resistance in Salmonella sv Enteritidis. Authors prepared adaptation media by adding acetic acid to TSB to different concentrations, and further adjusting the media pH to designated levels, by which they calculated the concentration of undissociated acetic acid for each acetate/pH combination. A S. Enteritidis strain grown in these adaptation media was challenged at pH 2.5 and the survival kinetics was used to assess the effect of adaptation on acid resistance. Authors also used a Greek acidic food, Tarama, to evaluated the effect of the adaptation on acid resistance. In addition, different acidulants and temperature were examined for the survival of acid adapted Salmonella.*

*The research objectives and the approaches seem reasonable. The outcomes are mixed. The adaptation in some acetate/pH combination (hence different undissociated acetic acid concentrations) seemed to enhance acid resistance, extending the time of 4 log reduction by few minutes when challenged at pH2.5. But this reviewer failed to see a clear trend for this effect. The adaptation did not affect the survival of S. Enteritidis in Tarama.*

*The presentation needs lot more polishing before ready for submission. Overall (especially so far for the methods and results sections), it reads like a graduate student’s earlier draft without too much input from the professor(s). Besides the numerous grammar and wording errors, the manuscript is somewhat disconnected among different sections. Some parts, especially the methods and results sections, need to be better delineated.*

**Authors’ reply: In the present study we primarily aimed to assess the impact of different amounts of undissociated acetic acid added over a range of different pH values on the induced acid resistance of *Salmonella* Enteritidis. We therefore adapted the pathogen to acid treatments and then challenged the cells at pH 2.5 (screening assay). The log transformed data of the microbiological analysis were used to assess the induction of acid resistance. We also used inactivation kinetic parameters (4D, i.e the time required for a four-log reduction) in order to further support our results. The inactivation kinetic data alongside with the log transformed data were statistically compared using appropriate homogeneity tests (Tukey’s HSD). A higher acid resistance was manifested for those treatments that significantly increased (P<0.05) both the enumerated log counts and the 4D estimates. All sections of the manuscript were thoroughly and extensively revised in order to present our findings in a more explicit and intelligible way. We also tried to keep a continuous flow in the text and among the different sections, in line with the reviewers’ comment. Any grammar and wording error that came to our notice was corrected.**

*L1. Delete “Studying for”. Overall, the title does not seem to capitulate the essence of the manuscript. Besides pH, what other food matrix associated factors were examined?*

**Authors’ reply: The effect of two intrinsic (pH value and acidulant of tarama salad) and one extrinsic (storage temperature) food associated factors were tested in culture media (TSB). pH adjustment of TSB was performed using either HCl in order to assess the effect of pH value of the food substrate or citric acid (i.e. the acidulant present in tarama salad) to examine whether the different acidulants could stimulate different adaptive responses. All broth samples were incubated either at 37^o^C (temperature of the screening assay) or 5^o^C (temperature where tarama salad was stored) (temperature effect). Please see lines 184-209, 511-584 of the marked copy and 162-185, 377-426 of the clear copy.**

**The title was rewritten in order to better encapsulate the essence of this study.**

*L60. What does “composition” mean here?*

**Authors’ reply: The term “composition” is initially referred to the nutrient composition of both adaptation and challenge media. It has been found that rich adaptation media promote higher acid survival compared to the minimal media. Similarly, the presence of certain amino acids (arginine, lysine) in the challenge media enhance the ability of *Salmonella* spp. to endure lethal acid stress, due to the contribution of these compounds to the induced homeostatic systems of arginine/agmatine and lysine/cadaverine. In addition, the presence of different acidulants in the adaptation medium may affect the magnitude of the induced acid resistance. Finally, acidification of the challenge substrate with different acidulants may, also, affect the acid resistance, with some acids being more potent in controlling foodborne pathogens.**

*L70. “the gastrointestinal human or animal track”?*

**Authors’ reply: The presence of certain organic acid, such as acetate, propionate, butyrate and valeric in the rumen or in the cecum of chickens has been reported. These organic acids are usually termed as Short Chain Fatty Acids and they are produced in milimollar quantities *via* fermentation of the feed carbohydrates. Indicatively, we cite the following references:**

- **Ricke S.C. 2003. Perspectives on the use of organic acids and short chain fatty acids as antimicrobials. Poultry Science 82: 632-639**
- **den Besten Gijs, van Eunen Karen, Groen Albert K., Venema Koen, Reijngoud Dirk-Jan, Bakker Barbara M. 2013. The role of short-chain fatty acids in the interplay between diet, gut microbiota and host energy metabolism. Journal of Lipid Research 54: 2325-2340**
- **Shen Hong, Xu Zhihui, Shen Zanming, Zhongyan Lu. 2019. The regulation of ruminal short-chain fatty acids on the functions of the rumen barriers. Frontiers in Physiology 10:1305**

*L71-72. Despite their antibacterial activity, pathogens have….”. What does “their” refer to? I believe authors mean organic acids, but in this sentence, it would mean pathogens.*

**Authors’ reply: The word “their” is replaced by “organic acids” (line 80 of the marked copy and 73-74 of the clear copy).**

*L79. “it is obvious that consumer safety renders questionable”. Very unclear. What does consumer safety render questionable? Or what does render consumer safety questionable?*

**Authors’ reply: All the sentence was deleted.**

*L82. What is the “drastic form” of an acid?*

**Authors’ reply: The term “drastic form” was used to refer to the molecules responsible for the antimicrobial effect of organic acids. This term was removed.**

*L86. “This serotype was selected as the most prevalent in Europe”. Change “as” to “as it was”, or “because it was”, etc.*

**Authors’ reply: The suggested correction “as it was” was adopted in the manuscript (line 596 of the marked copy and 437 of the clear copy).**

*L88. It does not seem “transferability” is the right word.*

**Authors’ reply: The word “transferability” was removed from the manuscript and all the sentences were rephrased appropriately.**

*L94. There is only a single strain. Also, change the order to Bacteria strain and growth conditions.*

**Authors’ reply: All the suggested corrections were fully accepted (line 119 of the marked copy and 99 of the clear copy).**

*L97-98, 100-101, etc. What does LabM etc mean? If that was the provider information, use the common format, as name, city, country). State incubation when giving temperatures and time information. Lab book information is not needed in the manuscript.*

**Authors’ reply: Provider information (name, city, country) was included in the Manuscript, in line with the suggested correction.**

**Time and temperature information regarding the incubation of TSA plates was excluded from the manuscript. Nevertheless, it has been shown that growth temperature and growth (stationary *vs* log) phase of the cells are important factors determining the acid resistance phenotypes of *Salmonella* spp.. Therefore, we feel that growth conditions of the strain must be included in the text. Please see lines 124-130 of the marked copy and 104-110 of the clear copy.**

*L104 etc. Capitalizer “ringer”*

**Authors’ reply: The suggested correction was accepted.**

*L117-144. This section talks about several things. May need additional subheadings to better delineate the procedures.*

**Authors’ reply: This section has been thoroughly revised, taken into consideration the reviewer comments above. Please see lines 148-291 of the marked copy and 127-214 of the clear copy.**

*L125. The use of “in vitro” or “in situ” etc is confusing and technically wrong. Suggest to avoid.*

**Authors’ reply: The terms *“in vitro”* and *“in situ”* have been removed from the manuscript.**

*L170, 179. Should “4” be 40?*

**Authors’ reply: *Salmonella* Enteritidis cells were resuspended in 4 ml of diluted tarama salad prior to inoculation to the food matrix. Diluted tarama salad was prepared by mixing 10 g of tarama with 30 g ¼ Ringer solution. The sentence has been rewritten to convey the message more explicitly. Please see lines 226-227 of the marked copy and 202-203 of the clear copy.**

*L183. What was done to make sure that those enumerated were S. enteritidis colonies on TSA plate, especially considering that the same plate was used for enumerating native microbiota (L191)?*

**Authors’ reply: Prior to inoculation, the indigenous microflora of each and every tarama salad package was determined using a general medium (TSA supplemented with sodium pyruvate). No native microbiota was detected. In order to further confirm that the enumerated colonies are *Salmonella*, plates corresponding to counts equal to or lower than 3.0-3.5 log CFU/g were selected and all colonies were streaked to Xylose Lysine Decarboxylase (XLD), a selective for *Salmonella* spp. medium.**

*L188, 190. Change “inherent” to indigenous, as in discussion.*

**Authors’ reply: The suggested correction was accepted (line 218 of the marked copy and 194 of the clear copy).**

*L220-222. If MIC was 5.2-7.2, and media with undissociated acid>7.2 mM were bactericidal, how was the adaptation in these media achieved? What was the initial inoculation in the adaptation media?*

**Authors’ reply: The populations inoculated in the adaptation media were approximately 6.5-7.0 log CFU/ml (lines 170-171 of the marked copy and 148-149 of the clear copy), whereas those recovered at the end of adaptation period were 6.1-6.9 log CFU/ml (lines 328-329 of the marked copy and 247-248 of the clear copy). Therefore, the bacterial counts remained almost constant during adaptation. This may be attributed to the short duration of adaptation (90 minutes at 37^o^C).**

*L290-291. “(6.9 mM, treatment 45mM/pH6.0)” Is this correct?*

**Authors’ reply: The sentence was corrected to “6.9 mM, treatment 45mM/pH5.5 (line 452 of the marked copy and 329 of the clear copy).**

*L312-314. Does this mean some of the values are from one model, while others from another model?*

**Authors’ reply: Weibull with tail model (Albert) is considered the extension of Weibull model that can incorporate shoulder and/or tailing phenomena if they are encountered. Therefore, these two models are comparable to each other. We initially used both equations for fitting the data of either screening assay (inactivation at TSB_2.5_) or tarama salad inactivation. However, after careful consideration, we decided to use Weibull with tail (Albert) model for the screening assay since it can better describe the obtained inactivation data. Similarly, Weibull model was used for fitting the data of tarama salad inactivation curves. In any case, the trends remained the same, with no substantial changes in the estimated values. We also included Root Mean Square Error (RMSE) parameter as an informative measure of goodness-of- fit. Please see Tables 1 and 2 and lines 293-308 of the marked copy and 216-229 of the clear copy.**

*L317. Change “independently” to regardless?*

**Authors’ reply: The suggested correction was accepted. The sentence was transferred with modifications to the Discussion section (line 645 of the marked copy and 477 of the clear copy).**

*L366. “at 4mM/pH6.0”. Is this correct?*

**Authors’ reply: The treatment was changed to 45mM/pH6.0 (line 641 of the marked copy and 475 of the clear copy).**
